# An efficient approach for detecting atrial fibrillation in ischemic stroke patients using a wearable device: a prospective multicenter substudy of the STABLED trial

**DOI:** 10.3389/fneur.2025.1560495

**Published:** 2025-08-13

**Authors:** Tomonari Saito, Yasuhiro Nishiyama, Toshiaki Otsuka, Yuki Sakamoto, Seiji Okubo, Yasuyuki Iguchi, Keiji Yamaguchi, Yasushi Okada, Hirotoshi Hamaguchi, Toshiro Yonehara, Masayuki Fukuzawa, Atsushi Takita, Takehiro Katano, Kazumi Kimura

**Affiliations:** ^1^Department of Neurology, Graduate School of Medicine, Nippon Medical School, Tokyo, Japan; ^2^Division of Medicine, Department of Neurology, Faculty of Medical Sciences, University of Fukui, Fukui, Japan; ^3^Department of Hygiene and Public Health, Graduate School of Medicine, Nippon Medical School, Tokyo, Japan; ^4^Department of Cerebrovascular Medicine, NTT Medical Center Tokyo, Tokyo, Japan; ^5^Department of Neurology, The Jikei University School of Medicine, Tokyo, Japan; ^6^Department of Neurology, Ichinomiya Nishi Hospital, Aichi, Japan; ^7^Department of Cerebrovascular Medicine and Neurology, National Hospital Organization Kyushu Medical Center, Fukuoka, Japan; ^8^Department of Neurology, Kita-Harima Medical Center, Hyogo, Japan; ^9^Department of Neurology, Saiseikai Kumamoto Hospital, Kumamoto, Japan; ^10^Primary Medical Science Department, Daiichi Sankyo Co., Ltd., Tokyo, Japan; ^11^Data Intelligence Department, Daiichi Sankyo Co., Ltd., Tokyo, Japan

**Keywords:** atrial fibrillation, ischemic stroke, wearable device, multicenter study, prospective study

## Abstract

**Objective:**

Stroke caused by atrial fibrillation (AF) is associated with high mortality and severe morbidity. Screening patients for AF may facilitate early initiation of anticoagulant therapy and prevent recurrent stroke; therefore, strategies to effectively detect AF in stroke patients are important.

**Methods:**

This prospective multicenter study was conducted between April 27, 2020 and March 31, 2021 at seven sites in Japan, as a substudy of the STABLED trial, a multicenter prospective randomized study to evaluate the efficacy and safety of catheter ablation with anticoagulant therapy using edoxaban in patients with ischemic stroke and AF. This substudy included 241 patients who suffered ischemic stroke but had no diagnosis of AF. Patients were monitored with Duranta, a wearable non-invasive wireless patch ECG system. The primary outcome was the detection rate for AF while wearing Duranta.

**Results:**

Of the 241 patients, 66.8% were men, and the mean age was 71.0 years. AF was detected in 21 of the 241 patients (8.7, 95% CI: 5.4–12.4) during follow-up using the Duranta wearable ECG system. ECG data were recorded for 7 days in all patients. The median number of days from stroke onset to Duranta placement was 2.0, but this duration varied considerably (median; IQR, 0–22.0). An adverse event of dermal pruritus was observed in 1 of the 241 patients (0.4%). Determinants for the detection of AF in patients with no previous history of AF were dyslipidemia and left atrial dimension.

**Conclusion:**

Wearable wireless patch ECG systems such as Duranta are simple and efficient devices for detecting AF. In patients with ischemic stroke and no diagnosis of AF, their use for detecting new AF may provide benefit through early initiation of anticoagulants and prevention of recurrent stroke.

## 1 Introduction

Stroke is the fourth leading cause of death and long-term disability in Japan (1). Cardiogenic embolic stroke is mainly caused by atrial fibrillation (AF), which is a primary risk factor for ischemic stroke (2). AF-associated stroke is often severe, and is associated with high mortality and severe morbidity. In addition, because the risk of recurrent cardiogenic embolic stroke caused by AF can be significantly reduced by anticoagulants (3, 4), the detection of AF in patients with acute ischemic stroke is important. However, AF is often absent on admission, as well as being paroxysmal or asymptomatic, making it difficult to detect with symptom-driven monitoring strategies. Therefore, several methods have been used to detect AF. Standard methods include 12-lead electrocardiography (ECG), Holter ECG, and long-term monitoring. However, because AF often still goes undetected, subcutaneous electrocardiographs are sometimes implanted in the patient’s chest, but these subcutaneous implantable devices are invasive, and there is a high demand for non-invasive methods that perform better in the detection of AF than the Holter-type ambulatory ECG. Currently, non-invasive devices, such as the ZIO patch (5), mobiCARE-MC100 TM (6), and AliveCor device (7), can effectively detect AF, although their use is not widespread. In addition, there are a number of non-regulatory consumer-based healthcare products that are capable of detecting AF, such as the Apple Watch series, although these are not certified for medical use, and their accuracy is insufficiently documented (8).

The STroke secondary prevention with catheter ABLation and EDoxaban (STABLED) trial in patients with non-valvular AF is a multicenter prospective randomized ongoing clinical trial to evaluate the efficacy and safety of catheter ablation with anticoagulant therapy using edoxaban in patients with AF and a history of recent ischemic stroke (9). In this substudy to the clinical trial, we took advantage of the data recorded from a particular cohort of patients who had recently suffered ischemic stroke (within 6 months) but had no history of non-valvular AF, and who were monitored for 7 days post-stroke using a relatively new device called Duranta, which is an iPhone-based single-lead electrocardiographic capture system (ZAIKEN, Co. Ltd., Tokyo, Japan). ECG data captured by the system are automatically transmitted to a cloud server via a dedicated iPhone. Duranta is non-invasive and can be easily attached and removed during hospitalization. The primary aim of this study was to evaluate the potential of a wearable ECG device for detecting AF in patients who had recently suffered ischemic stroke, and not to make direct comparisons between different devices.

In this companion study, we evaluated the detection rate of AF during hospitalization and the number of days when AF was most frequently detected by the Duranta system in the STABLED candidate stroke patients without a diagnosis of AF. Moreover, we made further analyses comparing clinical factors in the group in which AF was detected with those in the non-detection group.

## 2 Materials and methods

### 2.1 Study design

This multicenter prospective study was conducted between April 27, 2020 and March 31, 2021 at seven sites in Japan. A list of all participating institutions and their investigators is provided in Supplementary Table S1.

**Figure 1:**
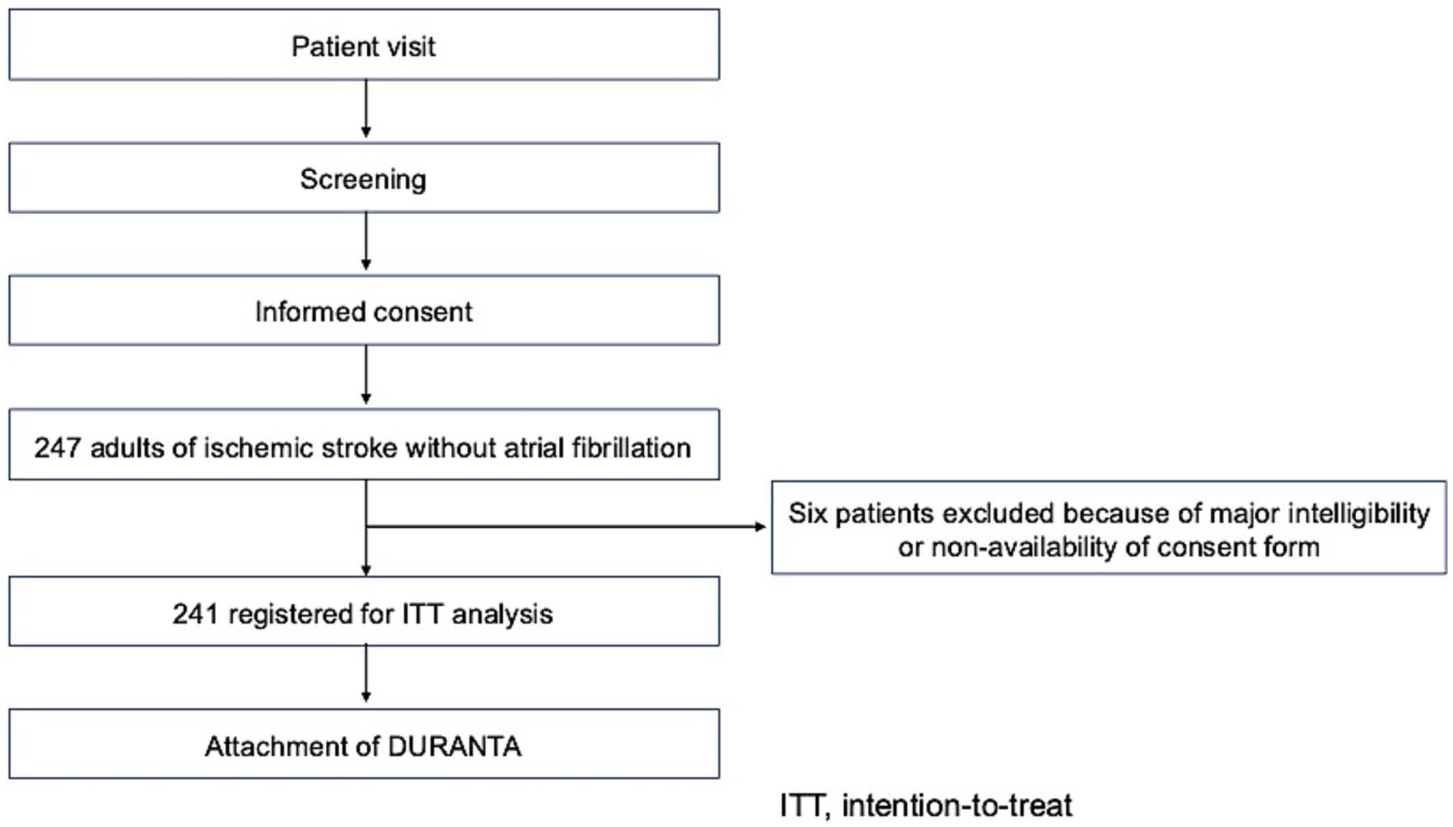
Flow chart of patient enrollment and screening.

The protocol was approved by the Nippon Medical School Certified Clinical Research Review Board (approval no. CRB3180001) and prospectively registered with the Japan Registry of Clinical Trials (registration no. jRCT s032200054; https://jrct.niph.go.jp/latest-detail/jRCTs032200054). The study was conducted in accordance with the Declaration of Helsinki and the Clinical Trials Act in Japan. All study participants provided written informed consent before enrollment. The study design is summarized in Figure 1.

### 2.2 Study participants

The eligible patients were candidates for the STABLED trial. The study patients were required to satisfy all of the defined inclusion criteria and none of the exclusion criteria. Briefly, the inclusion criteria were: (1) age ≥20 and ≤85 years at the time of informed consent, (2) no history of non-valvular AF, (3) history of ischemic stroke in the previous 6 months, and (4) a modified Rankin Scale ≤3 or expected to improve to ≤3 with treatment. The major exclusion criteria were: (1) severe renal impairment (creatinine clearance rate <30 mL/min), (2) markedly reduced cardiac function (ejection fraction <35%), (3) atrial septal defect, (4) being unlikely to complete the study because of progressive malignancy, (5) participation or planning to participate in another interventional clinical trial, and (6) otherwise judged as not being suitable for the study by the investigators.

### 2.3 Duranta: single-lead ECG monitor

**Figure 2:**
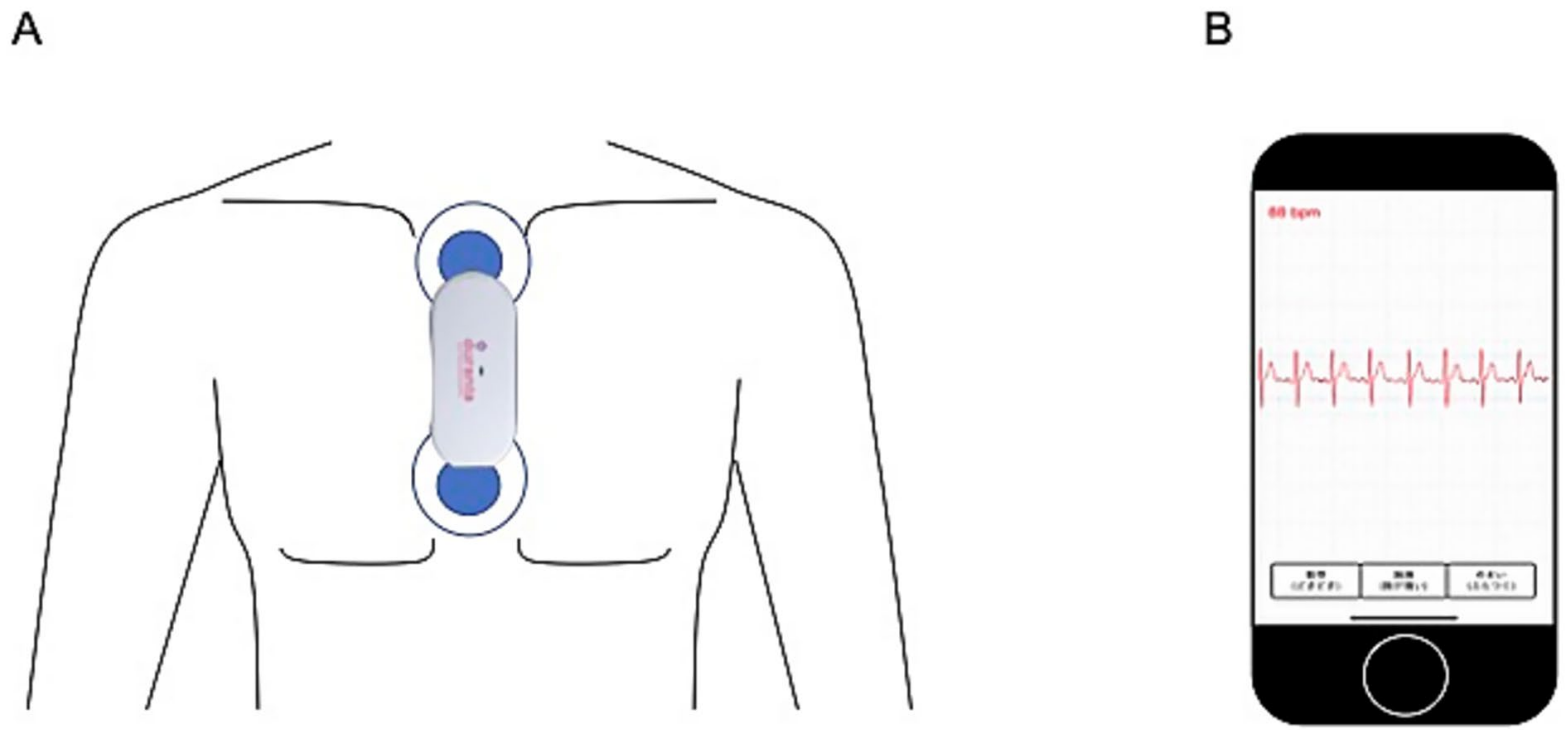
The Duranta wireless patch-type electrocardiographic monitoring system. The monitor is placed in a precordial position with a pair of electrode patches **(A)**. Recorded waveform data are sent to a bedside iPhone **(B)** via Bluetooth and then to a cloud server. Real-time ECG data can be checked on the iPhone. Physicians or medical staff can access the data on cloud server from PCs in the hospital for real-time observation.

The Duranta wireless non-invasive patch ECG monitoring system (Medical Device Certificate Number 226AIBZX00055000) is a small and lightweight device (78.4 mm wide × 35.1 mm deep × 14.7 mm thick, 35 g) with two patch electrodes; the device is placed in a precordial position (Figure 2A). The battery lasts for up to 7 consecutive days without recharging. ECG data are automatically transmitted to a cloud server via a dedicated iPhone (Figure 2B). Medical staff can access the cloud server with a personal ID and password, view the patient’s ECG in real time on an iPad, and download the ECG data to a computer in the hospital. For this study, ECG recording was started immediately after the main switch was turned on, and the recorded waveform data were sent to a nearby iPhone via Bluetooth, and then further relayed to a cloud server via a 3G/4G/LTE/Wi-Fi network. The patients’ personal information was not entered into the ECG devices, and there was therefore no risk of personal data leakage during the data delivery from the ECG devices.

### 2.4 Data collection

The following clinical information was obtained from the study data set: age, sex, ethnicity, body mass index, hypertension, dyslipidemia, diabetes, chronic kidney disease, congestive heart failure, and history of stroke or transient ischemic attack. In addition, current smoking, dementia, drinking habits, malignancy (active), modified Rankin Scale score, and National Institutes of Health Stroke Scale (NIHSS) score at baseline were assessed. The cardiothoracic ratio and brain natriuretic peptide (BNP) or N terminal (NT)-proBNP were also measured. Elevated BNP and NT-proBNP levels were defined as BNP > 100 pg./mL and NT-proBNP > 400 pg./mL (10). Transthoracic echocardiography measurements were performed at baseline by sonographers at each medical institution. The left ventricular ejection fraction and left atrial dimension (LAD) were evaluated. This study included patients with symptomatic ischemic stroke identified on magnetic resonance imaging (MRI) and diagnosed by Japanese stroke specialists. Diagnoses of hypertension, dyslipidemia, diabetes, renal dysfunction, and congestive heart failure were made by the responsible physicians at each site based on the results of medications, blood tests, and chest X-rays.

### 2.5 Outcomes

The primary outcome was the AF detection rate while wearing Duranta. Exploratory outcomes included the number of days from Duranta placement to AF detection, number of days from stroke onset to AF detection, AF duration time, number of days from stroke onset to Duranta placement, number of AF events detected during Duranta placement, and duration of Duranta placement. Safety outcomes included any adverse events due to Duranta placement.

### 2.6 Statistical analyses

Continuous variables are expressed as median (interquartile range [IQR]), or number (%). Categorical data are expressed as the number of subjects (%). For comparisons of baseline characteristics, clinical characteristics, and examination-related factors between patients in whom Duranta detected AF and those in whom it did not, the chi-squared (χ^2^) test was used for categorical variables, and the Mann–Whitney test was used for continuous variables. Univariate analysis and multivariate logistic regression analysis were performed to identify factors (demographics, comorbidities, laboratory findings, physical examinations, age, and sex) associated with the detection of AF, with variables demonstrating *p* < 0.05 in the univariable test being entered into the multivariate logistic regression. Statistical significance was set at *p* < 0.05. All analyses were performed with SPSS version 27.0 (IBM, Armonk, NY), while the 95% confidence intervals for AF detection rates were calculated using Stata version 17 (StataCorp LLC, College Station, TX).

## 3 Results

### 3.1 Baseline characteristics of the patients

A total of 241 patients were enrolled in this study (161 men, 80 women). The median patient age was 71.0 years (IQR: 62.5–78.0) for all patients, 71.0 years (IQR: 62.0–78.0) for patients without AF (AF−), and 73.0 years (IQR: 67.0–79.0) for patients with AF (AF+). The clinical characteristics of the patients are summarized in Table 1. Dyslipidemia (AF−: 120 patients [54.5%], AF+: 17 patients, [81.0%], *p* = 0.02), cardiothoracic ratio (AF−: 51.0 [IQR 46.9–55.9], AF+: 56.0 [IQR 52.2–57.0], *p* = 0.003), and LAD (AF−: 34.1 [IQR 31.0–39.0], AF+: 36.1 [IQR 33.8–44.5.], *p* = 0.032) were significantly different between the groups (Table 1).

**Table 1:** Baseline characteristics of the study subjects (*n* = 241).

	ALL	AF (−)	AF (+)	*p* value
Subjects, *n*	241	220	21	
Age, median (IQR), years	71.0 (62.5–78.0)	71.0 (62.0–78.0)	73.0 (67.0–79.0)	0.114
Sex				
Male, *n* (%)	161 (66.8)	150 (68.2)	11 (52.4)	0.152
Body-mass index, median (IQR), kg/m2	23.0 (21.0–25.5)	23.0 (21.0–25.4)	23.8 (22.1–27.0)	0.126
Comorbidities, *n* (%)	241	220	21	
Hypertension	167 (69.3)	153 (69.5)	14 (66.7)	0.786
Diabetes	63 (26.1)	56 (25.5)	7 (33.3)	0.443
Dyslipidemia	137 (56.8)	120 (54.5)	17 (81.0)	0.015
Chronic kidney disease	6 (2.5)	5 (2.3)	1 (4.8)	0.425
Congestive heart failure	3 (1.2)	3 (1.4)	0 (0)	1.000
Previous stroke/TIA	50 (20.8)	46 (20.9)	4 (19.0)	0.839
Malignant tumor	9 (3.7)	8 (3.6)	1 (4.8)	0.802
Current smoker	59 (24.5)	55 (25.0)	4 (19.0)	0.577
Drinking habit	63 (26.1)	58 (26.4)	5 (23.8)	0.797
Clinical scores and labs, *n*	241	220	21	
mRS score at admission	1 (1–3)	1 (1–3)	1.5 (1.0–3.0)	0.867
NIHSS score at admission	2 (1–4)	2 (1–4)	3 (1–13)	0.245
CTR (%)	51.5 (47.0–56.0)	51.0 (47.0–55.8)	56.0 (52.3–57.0)	0.003
LVEF (%)	65.7 (61.0–71.0)	66.0 (61.0–71.0)	64.0 (61.6–68.0)	0.649
LAD (mm)	34.8 (31.0–39.0)	34.1 (31.0–39.0)	36.1 (34.0–44.0)	0.032
BNP or NT-proBNP elevated	40 (16.6)	36 (16.4)	4 (19.0)	0.756
Acute revascularization, *n* (%)				
tPA	26 (10.8)	22 (10.0)	4 (19.0)	0.238
MT	31 (12.9)	26 (11.8)	5 (23.8)	0.149
Stroke etiology, *n* (%)				
Small vessel disease	57 (23.7)	52 (23.6)	5 (23.8)	0.881
Large-artery atherosclerosis	31 (12.9)	29 (13.2)	2 (9.5)	
Others	153 (63.5)	139 (63.2)	14 (66.7)	

AF, atrial fibrillation; BNP, brain natriuretic peptide; CTR, cardiothoracic ratio; IQR, interquartile range; LAD, left atrial dimension; LVEF, left ventricular ejection fraction; MT, mechanical thrombectomy; mRS, modified Rankin scale; NIHSS, National Institutes of Health Stroke Scale; NT, N-terminal; tPA, tissue plasminogen activator; TIA, transient ischemic attack.

### 3.2 Primary and exploratory outcomes

AF, the primary outcome for this study, was detected in 21 of the 241 patients (8.7, 95% CI: 5.4–12.4) during follow-up using Duranta. The median number of days from Duranta placement to first AF detection was 2.0 (IQR: 1.0–3.5), the median AF duration time was 59 min (IQR: 7.5–397.5), the median number of AF detections in the AF + group was 2.0 (IQR: 1.0–3.5), the median number of days from stroke onset to AF detection was 4.0 (IQR: 3.0–6.0), and the duration of Duranta placement was 7 days in all patients (Table 2).

**Table 2:** Primary and exploratory outcomes.

Primary outcomes	
Detection rate of AF while wearing Duranta, *n*, (%)	21 (8.7, 5.4–12.4)
Exploratory outcomes	
Number of days from stroke onset to Duranta placement, median (IQR)	2.0 (0–22.0)
Number of days of Duranta placement, median (IQR)	7.0 (7.0–7.0)
Number of days from Duranta placement to first AF detection, median (IQR)	2.0 (1.0–3.5)
Number of days from stroke onset to AF detection, median (IQR)	4.0 (3.0–6.0)
AF duration time, median (IQR), minutes	59 (7.5–397.5)
Number of AF events detected, median (IQR)	2.0 (1.0–3.5)

AF, atrial fibrillation; IQR, interquartile range.

### 3.3 Adverse events

An adverse event occurred in only 1 (0.4%) of the 241 patients during Duranta placement. This was a case of dermal pruritus.

### 3.4 Time from stroke onset to Duranta placement in 21 patients with AF detected

**Figure 3:**
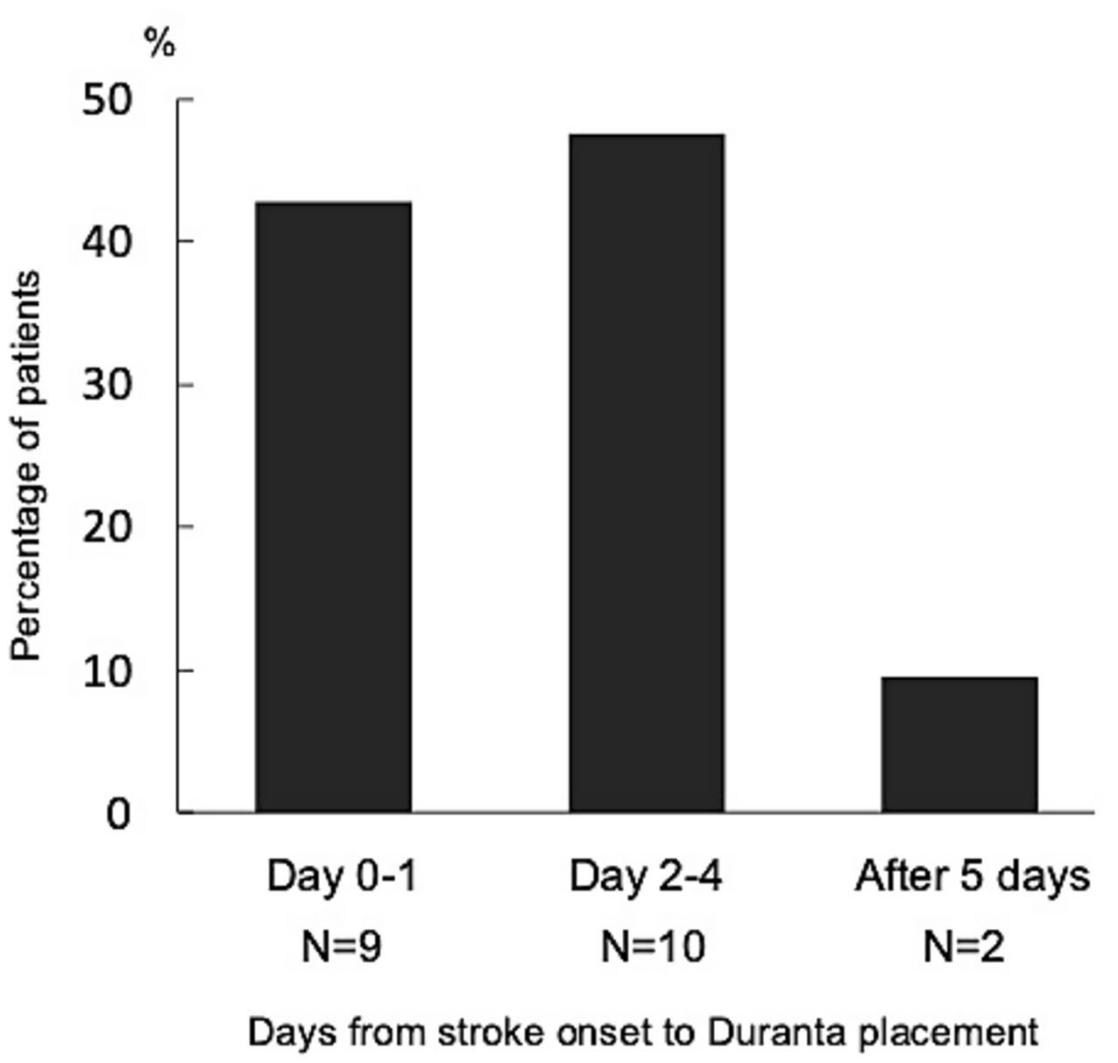
Time from stroke onset to Duranta was placed within 1 day after stroke onset in 9 patients (42.9%) of 21 patients with AF detected, between 2 and 4 days after stroke onset in 10 patients (47.6%), and 5 days or more after stroke onset in 2 patients (9.5%).

Patients could be enrolled up to 6 months after stroke onset, and therefore Duranta was not always placed promptly after admission. Among the 21 patients in whom AF was detected, the median number of days from stroke onset to Duranta placement was 2.0 (IQR: 1.0–22.0), with 41.9% being equipped with Duranta 1 day after stroke onset, 47.6% between 2 and 4 days after stroke onset, and 9.5% 5 days or more after stroke onset (Figure 3).

### 3.5 Probability of new AF within the duration of Duranta placement

**Figure 4:**
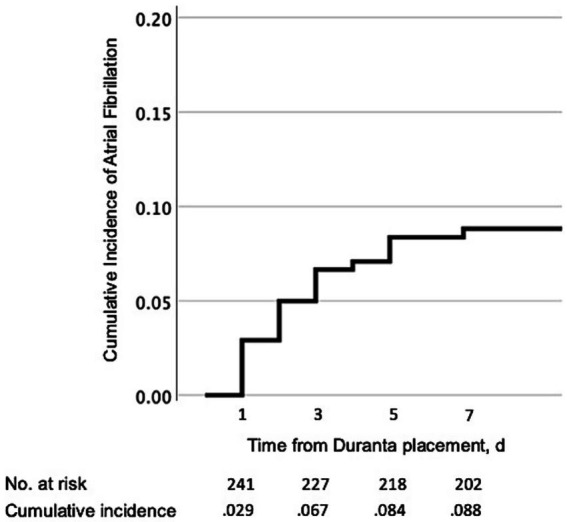
Kaplan–Meier curve for the cumulative incidence of detection of new atrial fibrillation with Duranta. The cumulative incidence rates at days 3 and 7 were 0.067 and 0.088, respectively. Most cases were detected within 3 days.

According to the Kaplan–Meier curve, the cumulative incidence rates for detection of AF were 0.029, 0.067, 0.084, and 0.88 at days 1, 3, 5, and 7 of Duranta placement, respectively (Figure 4).

### 3.6 Determinants of AF detection with Duranta

In the univariable analysis, dyslipidemia, NIHSS score at admission, cardiothoracic ratio, and LAD were identified as determinants of AF detection using Duranta. In the multivariable analysis, we adjusted for multiple potential confounders such as age, sex, dyslipidemia, NIHSS score at admission, cardiothoracic ratio, and LAD, and only dyslipidemia (adjusted odds ratio [aOR]: 3.92, 95% CI: 1.17–13.13, *p* = 0.027) and LAD (aOR: 1.10, 95% CI: 1.01–1.20, *p* = 0.029) were significantly associated with AF detection by Duranta (Table 3).

**Table 3:** Determinants of atrial fibrillation detection with DURANTA.

	Univariable		Multivariable	
	OR (95%CI)	*P* value	OR (95%CI)	*P* value
Age	1.04 (0.99–1.09)	0.114	1.04 (0.96–1.07)	0.604
Sex				
Female	Ref			
Male	0.51 (0.21–1.27)	0.147	0.72 (0.25–2.10)	0.557
Body-mass index	1.12 (0.99–1.26)	0.066		
Comorbidities				
Hypertension	0.88 (0.34–2.27)	0.785		
Diabetes	1.46 (0.56–3.81)	0.435		
Dyslipidemia	3.54 (1.15–10.87)	0.027	3.92 (1.17–13.13)	0.027
Chronic kidney disease	2.15 (0.24–19.31)	0.494		
Congestive heart failure	NA*			
Previous stroke/TIA	0.89 (0.29–2.77)	0.841		
Malignant tumor	1.33 (0.16–11.14)	0.796		
Current smoker	0.76 (0.42–1.35)	0.348		
Drinking habit	0.87 (0.31–2.49)	0.799		
Clinical scores and laboratory analyses				
mRS score at admission	1.06 (0.74–1.51)	0.763		
NIHSS score at admission	1.06 (1.01–1.12)	0.030	1.05 (0.99–1.12)	0.107
CTR	1.11 (1.03–1.20)	0.006	1.07 (0.97–1.18)	0.173
LVEF	1.00 (0.95–1.06)	0.969		
LAD	1.11 (1.02–1.20)	0.014	1.10 (1.01–1.20)	0.029
BNP or NT-proBNP elevated	1.20 (0.38–3.78)	0.752		
Acute revascularization				
tPA	2.12 (0.65–6.86)	0.211		
MT	2.33 (0.79–6.90)	0.126		

*Not used as an explanatory variable because neither outcome was observed.

Variables of age, sex, and those showing *p* < 0.05 in the univariable analysis were included in the final multivariable model. BNP, brain natriuretic peptide; CTR, cardiothoracic ratio; LAD, left atrial dimension; LVEF, left ventricular ejection fraction; MT, mechanical thrombectomy; mRS, modified Rankin scale; NIHSS, National Institutes of Health Stroke Scale; NT, N-terminal; OR, odds ratio; tPA, tissue plasminogen activator; TIA, transient ischemic attack.

## 4 Discussion

This prospective multicenter study of patients with ischemic stroke in Japan investigated the detection of new atrial fibrillation with a non-invasive device, an iPhone-based single-lead electrocardiographic capture system.

When a patient with AF has a stroke, it is often severe (2) and the recurrence rate is high (11, 12), although anticoagulants are highly effective in preventing recurrence (3, 4). Therefore, when a patient without a history of AF has a stroke, tests to detect AF are routinely performed, usually ECG and Holter ECG (13). Compared with 24-h ECG recordings, 7-day Holter ECG recordings were shown to have better detection of AF (14, 15). Although implantable ECGs have been shown to provide a high level of evidence for the detection of AF after thorough evaluation (16, 17), insertable cardiac monitors are invasive to the patient and can cause adverse events, and early implantation after the onset of stroke is generally not performed. In addition, early rhythm control for AF improves the primary clinical outcome (18). Given the high rate of recurrent strokes in patients with AF, if a patient without a history of AF has a stroke and is subsequently found to have AF, prompt administration of anticoagulation to reduce the risk of recurrence and early intervention for rhythm control of AF are required. These require heart rate monitoring that is as simple and safe as possible for several days or longer, starting as early as possible after stroke onset.

This study enrolled patients without a known history of AF and without AF on the admission 12-lead ECG. If AF was observed on the admission ECG, the patient would have been diagnosed with cardioembolic stroke and excluded. Therefore, the study included patients with lacunar infarction, as well as those with cryptogenic stroke and embolic stroke of undetermined source (ESUS); however, at the time of admission, testing to determine the stroke etiology (e.g., transthoracic echocardiography, Holter ECG, transesophageal echocardiography, lower limb venous ultrasound, cerebral angiography, contrast-enhanced CT) was incomplete, so a definitive diagnosis of cryptogenic stroke or ESUS could not be made at that point. We can therefore only describe the patients as “ischemic stroke cases without AF on admission ECG.” We believe the population likely includes cases that would eventually receive a diagnosis of cryptogenic stroke or ESUS after further testing.

According to the European Society of Cardiology 2020 guidelines (19), single-lead ECG recording using a wearable device can be used to confirm the diagnosis of AF (recommendation: Ia). In addition, a recent systematic review and meta-analysis suggests that non-invasive rhythm monitoring strategies should precede invasive monitoring (20). Detection of AF with wearable electrocardiographs, including patch ECG devices and wristwatch health products, has been reported. Studies such as the Apple Heart Study (21) and SCREEN-AF (SCREENing for Atrial Fibrillation) (22) using a single-lead ECG have recently demonstrated the feasibility and effectiveness of single-lead ECG recorders for detecting AF after stroke. However, the results of these studies were based on data from the general population and were not specific to patients with ischemic stroke or transient ischemic attack (23). Wristwatch devices are simple and have the potential to screen many asymptomatic and symptomatic patients; however, these devices use a photoplethysmograph sensor to detect atrial fibrillation when checking heartbeats in the background (24), and they demonstrate variable sensitivity and specificity and do not have acceptable accuracy compared with prescribed ECG devices. In comparison, a patch ECG device usually uses a single channel of bipolar induction over the heart to record the ECG.

In this study, we used Duranta, a patch-type wireless real-time ECG monitor that is easy to attach to the patient. Duranta is iPhone-based, and one of the features of this system is that it is very easy to attach and remove if the patient has to go for other examinations such as CT, MRI, or echography, as well as to use the restroom or take a bath during their hospital stay.

The AF detection rate of the Duranta system was 8.7%, which is higher than the 4–5% (22, 25) reported in screening studies and the 5–7% (5, 26) in studies with a population of stroke patients. Furthermore, it is higher than the AF detection rate of 6.8% in a study using 7-day Holter monitoring in patients who suffered a recent embolic stroke of undetermined source (26). However, the data used in this study came from a subset of patients recruited to the STABLED trial who underwent monitoring with the Duranta system, and within the scope of this study, it is not possible to accurately compare the performance of the Duranta system in comparison with similar systems; other similar single-lead and patch portable ECG systems would likely provide similar results, with the evidence suggesting that the Duranta system is at least on a par with similar systems.

In this study, LAD and dyslipidemia were found to be associated with detection of AF after adjusting for confounding factors. Left atrial enlargement is well known to be an inducer of AF (27); the larger the left atrial size, the greater the risk of developing AF (28). Recently, prespecified analysis of the STROKE AF randomized clinical trials found that only congestive heart failure and left atrial enlargement were significant predictors of AF (29). Another study reported that not only left atrial enlargement, but also the left atrial volume index (LAVI), calculated by dividing the left atrial volume by the body surface area, was higher in an AF + group than in an AF− group (30). Unfortunately, because this was a multicenter registry study, echocardiography was not performed for all patients and LAVI was thus excluded from the statistical analysis. Therefore, LAD was used as a valuable clinical predictor for the detection of AF.

The finding of the association between dyslipidemia and AF appears to be complex and paradoxical. In a previous study, higher total cholesterol (TC) and low-density lipoprotein cholesterol (LDL-C) levels were inversely associated with the incidence of AF over a mean follow-up period of 7.12 years (31). Low high-density lipoprotein (HDL) cholesterol was associated with an increased risk of new-onset AF in women, but not in men (32). Low HDL-C levels and high triglyceride (TG) levels, which are markers of metabolic syndrome, are associated with a higher risk of AF (33, 34). Reports of dyslipidemia and AF remain inconclusive, although low HDL-C and high TG levels may be associated with AF because metabolic syndrome is reported to be associated with the risk for AF (35). However, as dyslipidemia was diagnosed by the physicians in this study, it is unclear which lipid fraction was abnormal.

This study has some potential limitations. First, we cannot generalize our findings to non-hospitalized patients because our study included only in-hospital patients with ischemic stroke. Second, this study was conducted only at Japanese sites, and therefore, it is unclear whether the results can be extrapolated to patients from other countries. Third, the number of participants included in the current epidemiological study was small, which may have affected our findings through a lack of statistical power. Fourth, the enrolled patients without a known history of AF and without AF on the admission 12-lead ECG included patients with lacunar infarction, as well as those with cryptogenic stroke and ESUS; however, at the time of admission, testing to determine the stroke etiology was incomplete, so a definitive diagnosis of cryptogenic stroke or ESUS could not be made at that point. Finally, for hypertension, dyslipidemia, diabetes, chronic kidney disease, and medical history, the presence or absence of comorbidities was recorded according to the physician in charge, and therefore information on oral medications and laboratory values was not available.

## 5 Conclusion

In this prospective multicenter study performed in Japan, we found that a non-invasive wireless patch ECG system, the iPhone-based single-lead electrocardiographic capture system Duranta, found AF in 21 out of 241 ischemic stroke patients without a previous history of AF. The median time from Duranta placement to first AF detection was 2 days. Determinants for AF detection in patients without a history of AF were dyslipidemia and LAD. In ischemic stroke patients with undiagnosed AF, the use of wireless patch ECG systems for detecting new AF may provide benefit in the form of early initiation of anticoagulant administration and prevention of recurrent stroke.

## Data Availability

The original contributions presented in the study are included in the article/Supplementary material, further inquiries can be directed to the corresponding author.

## References

[1] Tai S-Y Cheon S Yamaoka Y Chien Y-W Lu T-H. Changes in the rankings of leading causes of death in Japan, Korea, and Taiwan from 1998 to 2018: a comparison of three ranking lists. BMC Public Health. (2022) 22:926. doi: 10.1186/s12889-022-13278-7, PMID: 35538508 PMC9086411

[2] Kimura K Minematsu K Yamaguchi T. Atrial fibrillation as a predictive factor for severe stroke and early death in 15,831 patients with acute ischaemic stroke. J Neurol Neurosurg Psychiatry. (2005) 76:679–83. doi: 10.1136/jnnp.2004.048827, PMID: 15834026 PMC1739612

[3] Mazurek M Shantsila E Lane DA Wolff A Proietti M Lip GYH. Secondary versus primary stroke prevention in atrial fibrillation: insights from the Darlington atrial fibrillation registry. Stroke. (2017) 48:2198–205. doi: 10.1161/strokeaha.116.016146, PMID: 28679859

[4] Yoshimura S Koga M Sato S Todo K Yamagami H Kumamoto M. Two-year outcomes of anticoagulation for acute ischemic stroke with Nonvalvular atrial fibrillation - SAMURAI-NVAF study. Circ J. (2018) 82:1935–42. doi: 10.1253/circj.CJ-18-0067, PMID: 29863095

[5] Tung CE Su D Turakhia MP Lansberg MG. Diagnostic yield of extended cardiac patch monitoring in patients with stroke or TIA. Front Neurol. (2014) 5:266. doi: 10.3389/fneur.2014.00266, PMID: 25628595 PMC4290477

[6] Jung S Lee HA Kang IS Shin SH Chang Y Woo Shin D. Clinical implications of atrial fibrillation detection using wearable devices in patients with cryptogenic stroke (CANDLE-AF) trial: design and rationale. Front Cardiovasc Med. (2022) 9:837958. doi: 10.3389/fcvm.2022.837958, PMID: 35445088 PMC9013795

[7] Halcox JPJ Wareham K Cardew A Gilmore M Barry JP Phillips C. Assessment of remote heart rhythm sampling using the AliveCor heart monitor to screen for atrial fibrillation: the REHEARSE-AF study. Circulation. (2017) 136:1784–94. doi: 10.1161/circulationaha.117.030583, PMID: 28851729

[8] Hashimoto K Harada N Kasamaki Y. Can a patch electrocardiographic device be a leading actor for detecting atrial fibrillation? - diversifying electrocardiographic monitoring devices. Circ J. (2022) 86:189–91. doi: 10.1253/circj.CJ-21-0644, PMID: 34471069

[9] Sakamoto Y Nishiyama Y Iwasaki YK Daida H Toyoda K Kitagawa K. Design and rationale of the STroke secondary prevention with catheter ABLation and EDoxaban clinical trial in patients with non-valvular atrial fibrillation: the STABLED study. J Cardiol. (2019) 74:539–42. doi: 10.1016/j.jjcc.2019.06.002, PMID: 31337525

[10] Bozkurt B Coats AJS Tsutsui H Abdelhamid CM Adamopoulos S Albert N. Universal definition and classification of heart failure: a report of the Heart Failure Society of America, heart failure Association of the European Society of cardiology, Japanese heart failure society and writing Committee of the Universal Definition of heart failure: endorsed by the Canadian heart failure society, heart failure Association of India, Cardiac Society of Australia and new Zealand, and Chinese heart failure association. Eur J Heart Fail. (2021) 23:352–80. doi: 10.1002/ejhf.2115, PMID: 33605000

[11] Hart RG Coull BM Hart D. Early recurrent embolism associated with nonvalvular atrial fibrillation: a retrospective study. Stroke. (1983) 14:688–93. doi: 10.1161/01.str.14.5.688, PMID: 6658950

[12] Paciaroni M Agnelli G Falocci N Caso V Becattini C Marcheselli S. Early recurrence and cerebral bleeding in patients with acute ischemic stroke and atrial fibrillation: effect of anticoagulation and its timing: the RAF study. Stroke. (2015) 46:2175–82. doi: 10.1161/strokeaha.115.008891, PMID: 26130094

[13] Higgins P MacFarlane PW Dawson J McInnes GT Langhorne P Lees KR. Noninvasive cardiac event monitoring to detect atrial fibrillation after ischemic stroke: a randomized, controlled trial. Stroke. (2013) 44:2525–31. doi: 10.1161/strokeaha.113.001927, PMID: 23899913

[14] Jabaudon D Sztajzel J Sievert K Landis T Sztajzel R. Usefulness of ambulatory 7-day ECG monitoring for the detection of atrial fibrillation and flutter after acute stroke and transient ischemic attack. Stroke. (2004) 35:1647–51. doi: 10.1161/01.STR.0000131269.69502.d9, PMID: 15155965

[15] Carrarini C Di Stefano V Russo M Dono F Di Pietro M Furia N. ECG monitoring of post-stroke occurring arrhythmias: an observational study using 7-day Holter ECG. Sci Rep. (2022) 12:228. doi: 10.1038/s41598-021-04285-6, PMID: 34997171 PMC8741921

[16] Bernstein RA Kamel H Granger CB Piccini JP Sethi PP Katz JM. Effect of long-term continuous cardiac monitoring vs usual care on detection of atrial fibrillation in patients with stroke attributed to large- or small-vessel disease: the STROKE-AF randomized clinical trial. JAMA. (2021) 325:2169–77. doi: 10.1001/jama.2021.6470, PMID: 34061145 PMC8170544

[17] Sagris D Papanikolaou A Kvernland A Korompoki E Frontera JA Troxel AB. COVID-19 and ischemic stroke. Eur J Neurol. (2021) 28:3826–36. doi: 10.1111/ene.15008, PMID: 34224187 PMC8444875

[18] Kirchhof P Camm AJ Goette A Brandes A Eckardt L Elvan A. Early rhythm-control therapy in patients with atrial fibrillation. N Engl J Med. (2020) 383:1305–16. doi: 10.1056/NEJMoa2019422, PMID: 32865375

[19] Hindricks G Potpara T Dagres N Arbelo E Bax JJ Blomström-Lundqvist C. 2020 ESC guidelines for the diagnosis and management of atrial fibrillation developed in collaboration with the European Association for Cardio-Thoracic Surgery (EACTS): the task force for the diagnosis and management of atrial fibrillation of the European Society of Cardiology (ESC) developed with the special contribution of the European heart rhythm association (EHRA) of the ESC. Eur Heart J. (2021) 42:373–498. doi: 10.1093/eurheartj/ehaa612, PMID: 32860505

[20] Noubiap JJ Agbaedeng TA Kamtchum-Tatuene J Fitzgerald JL Middeldorp ME Kleinig T. Rhythm monitoring strategies for atrial fibrillation detection in patients with cryptogenic stroke: a systematic review and meta-analysis. Int J Cardiol Heart Vasc. (2021) 34:100780. doi: 10.1016/j.ijcha.2021.100780, PMID: 33948484 PMC8080458

[21] Perez MV Mahaffey KW Hedlin H Rumsfeld JS Garcia A Ferris T. Large-scale assessment of a smartwatch to identify atrial fibrillation. N Engl J Med. (2019) 381:1909–17. doi: 10.1056/NEJMoa1901183, PMID: 31722151 PMC8112605

[22] Gladstone DJ Wachter R Schmalstieg-Bahr K Quinn FR Hummers E Ivers N. Screening for atrial fibrillation in the older population: a randomized clinical trial. JAMA Cardiol. (2021) 6:558–67. doi: 10.1001/jamacardio.2021.0038, PMID: 33625468 PMC7905702

[23] Yan B Tu H Lam C Swift C Ho MS Mok VCT. Nurse led smartphone electrographic monitoring for atrial fibrillation after ischemic stroke: SPOT-AF. J Stroke. (2020) 22:387–95. doi: 10.5853/jos.2020.00689, PMID: 33053954 PMC7568969

[24] Liao MT Yu CC Lin LY Pan KH Tsai TH Wu YC. Impact of recording length and other arrhythmias on atrial fibrillation detection from wrist photoplethysmogram using smartwatches. Sci Rep. (2022) 12:5364. doi: 10.1038/s41598-022-09181-1, PMID: 35354873 PMC8967835

[25] Steinhubl SR Waalen J Edwards AM Ariniello LM Mehta RR Ebner GS. Effect of a home-based wearable continuous ECG monitoring patch on detection of undiagnosed atrial fibrillation: the mSToPS randomized clinical trial. JAMA. (2018) 320:146–55. doi: 10.1001/jama.2018.8102, PMID: 29998336 PMC6583518

[26] Miyazaki Y Toyoda K Iguchi Y Hirano T Metoki N Tomoda M. Atrial fibrillation after ischemic stroke detected by chest strap-style 7-day Holter monitoring and the risk predictors: EDUCATE-ESUS. J Atheroscler Thromb. (2021) 28:544–54. doi: 10.5551/jat.58420, PMID: 32801289 PMC8193782

[27] Goldberger JJ Arora R Green D Greenland P Lee DC Lloyd-Jones DM. Evaluating the atrial myopathy underlying atrial fibrillation: identifying the Arrhythmogenic and Thrombogenic substrate. Circulation. (2015) 132:278–91. doi: 10.1161/circulationaha.115.016795, PMID: 26216085 PMC4520257

[28] Tsang TS Barnes ME Bailey KR Leibson CL Montgomery SC Takemoto Y. Left atrial volume: important risk marker of incident atrial fibrillation in 1655 older men and women. Mayo Clin Proc. (2001) 76:467–75. doi: 10.4065/76.5.467, PMID: 11357793

[29] Schwamm LH Kamel H Granger CB Piccini JP Katz JM Sethi PP. Predictors of atrial fibrillation in patients with stroke attributed to large- or small-vessel disease: a Prespecified secondary analysis of the STROKE AF randomized clinical trial. JAMA Neurol. (2023) 80:99–103. doi: 10.1001/jamaneurol.2022.4038, PMID: 36374508 PMC9664367

[30] De Angelis MV Di Stefano V Franciotti R Furia N Di Girolamo E Onofrj M. Cryptogenic stroke and atrial fibrillation in a real-world population: the role of insertable cardiac monitors. Sci Rep. (2020) 10:3230. doi: 10.1038/s41598-020-60180-6, PMID: 32094376 PMC7040015

[31] Li X Gao L Wang Z Guan B Guan X Wang B. Lipid profile and incidence of atrial fibrillation: a prospective cohort study in China. Clin Cardiol. (2018) 41:314–20. doi: 10.1002/clc.22864, PMID: 29575115 PMC6490045

[32] Watanabe H Tanabe N Yagihara N Watanabe T Aizawa Y Kodama M. Association between lipid profile and risk of atrial fibrillation. Circ J. (2011) 75:2767–74. doi: 10.1253/circj.cj-11-0780, PMID: 21914959

[33] Sagris D Harrison SL Lip GYH. Lipids and atrial fibrillation: new insights into a paradox. PLoS Med. (2022) 19:e1004067. doi: 10.1371/journal.pmed.1004067, PMID: 35951513 PMC9371346

[34] Ding M Wennberg A Gigante B Walldius G Hammar N Modig K. Lipid levels in midlife and risk of atrial fibrillation over 3 decades-experience from the Swedish AMORIS cohort: a cohort study. PLoS Med. (2022) 19:e1004044. doi: 10.1371/journal.pmed.1004044, PMID: 35951514 PMC9371362

[35] Zheng Y Xie Z Li J Chen C Cai W Dong Y. Meta-analysis of metabolic syndrome and its individual components with risk of atrial fibrillation in different populations. BMC Cardiovasc Disord. (2021) 21:90. doi: 10.1186/s12872-021-01858-1, PMID: 33588759 PMC7885417

