# Supplementary material for: An efficient approach for detecting atrial fibrillation in ischemic stroke patients using a wearable device: a prospective multicenter substudy of the STABLED trial
Source: Front Neurol. 2025 Aug 13;16:1560495. doi: 10.3389/fneur.2025.1560495 (PMC12391382; doi:10.3389/fneur.2025.1560495)
Supplement: Supplementary file 1 [file Data_Sheet_1.pdf]

Supplementary Material

1 Supplementary Table

Table S1. List of all participating institutions and their investigators.

**Institution**	**Investigators**
Department of Neurology, Graduate School of Medicine, Nippon Medical School, Tokyo, Japan	Tomonari Saito; Yuki Sakamoto; Takehiro Katano; Kazumi Kimura
Division of Medicine, Department of Neurology, Faculty of Medical Sciences, University of Fukui, Fukui, Japan	Yasuhiro Nishiyama
Department of Hygiene and Public Health, Graduate School of Medicine, Nippon Medical School, Tokyo, Japan	Toshiaki Otsuka
Department of Cerebrovascular Medicine, NTT Medical Center Tokyo, Tokyo, Japan	Seiji Okubo
Department of Neurology, the Jikei University School of Medicine, Tokyo, Japan	Yasuyuki Iguchi
Department of Neurology, Ichinomiya Nishi Hospital, Aichi, Japan	Keiji Yamaguchi
Department of Cerebrovascular Medicine and Neurology, National Hospital Organization Kyushu Medical Center, Fukuoka, Japan	Yasushi Okada
Department of Neurology, Kita-Harima Medical Center, Hyogo, Japan	Hirotoshi Hamaguchi
Department of Neurology, Saiseikai Kumamoto Hospital, Japan	Toshiro Yonehara

Primary Medical Science Department, Daiichi Sankyo Co., Ltd., Tokyo, Japan	Masayuki Fukuzawa
Data Intelligence Department, Daiichi Sankyo Co., Ltd., Tokyo, Japan	Atsushi Takita
